# Supplementary material for: Are carbon emissions trading and green financial instruments synergistic? -Comprehensive quantitative research based on content analysis
Source: PLoS One. 2024 Mar 7;19(3):e0298601. doi: 10.1371/journal.pone.0298601 (PMC10919601; doi:10.1371/journal.pone.0298601)
Supplement: S1 Data — (ZIP) [file pone.0298601.s001.zip › Data/地级市Z_F工作报告.zip!/╣π╬≈/║╪╓▌╩╨/└┤╫╘╣1⁄2╓┌║┼í╛┬φ┐╦╩2╛▌═°í┐_2010.docx]

各位代表：

现在，我代表市人民政府向大会报告工作，请予审议，并请市政协委员和列席会议的同志提出意见。

一、2009年工作回顾

2009年，是我们在危机中抢抓机遇、在逆境中克难奋进的一年。受国际金融危机的严重冲击和影响，我市经济社会发展遇到极大困难，年初主要工业行业大面积亏损，近四分之一的规模以上企业停产，主导产品市场萎缩，价格下跌，利税缩减，企业效益下滑，严重影响财政收入，一季度连续三个月财政收入呈负增长，部分农产品价格大幅波动，农民工大量返乡，就业形势极其严峻。面对困难和挑战，在市委的坚强领导下，在市人大、市政协的全力支持下，市政府积极谋划，全力应对，紧紧围绕“四保”的目标任务，及时采取了一系列超常规措施，使我市逐步扭转了被动局面，全年经济逆势而上，实现了加速提质发展，社会大局保持了和谐稳定。具体表现在以下五方面：

——经济持续较快增长。预计全市生产总值280亿元，增长12.2%。财政收入18.15亿元，增长13.4%。其中，地方一般预算性收入10.39亿元，增长22%，增幅位居全区第7。实现工业增加值116.53亿元，增长7.9%；农业增加值58.9亿元，增长5.5%；工业技改投资51.6亿元，完成自治区下达任务的172%，增长194.8%，增幅位居全区第1。

——扩大内需取得突破。全社会固定资产投资254.68亿元，增长59.8%，人均固定资产投资首次突破1万元。其中，城镇固定资产投资212.82亿元，增长59.4%，增幅位居全区第3。社会消费品零售总额68.87亿元，增长18%。金融机构新增贷款总量和增幅均创历史新高，新增贷款38.37亿元，完成自治区下达任务的153.5%，增长52.8%，增幅位居全区第2。

——对外开放成效显著。外贸出口总额1.35亿美元，增长35%，增幅位居全区第3。全市招商引资新签项目投资总额240亿元，增长63%。其中，内资项目到位资金175亿元，增长81.1%，完成自治区下达任务的159%，增幅位居全区第5。

——环境质量不断提升。万元生产总值能耗下降3%，二氧化硫排放量控制在3.98万吨以内，化学需氧量排放量控制在3.2万吨以内。

——人民生活切实改善。新增就业13619人，城镇登记失业率控制在3.57%以内。城镇居民人均可支配收入14509元，增长13.6%；农民人均纯收入3876元，增长12.1%。人口自然增长率为9.27‰。

一年来，我们主要做了以下工作：

(一)突出投资拉动，项目建设实现重大突破。我们坚持把项目建设作为拉动增长最有效的手段，扎实开展“项目建设年”活动。年初，经市委决定，我市成立重点项目建设指挥部，认真落实领导联系重大项目制度，强化项目服务和督查，严格落实问责制，组织实施445个重点项目建设，总投资834亿元，年度完成投资177亿元，全年新开工项目253个，开工率98%。列入2009年自治区层面统筹推进新开工和预备开工项目8个全部开工或启动建设。争取中央扩大内需投资项目4批272项，完成投资4.8亿元。项目前期经费投入2000万元，超过建市以来前6年的总和。2009年是我市历年来项目建设规模最大、数量最多、完成投资最好的一年，对拉动经济回升和平稳增长起到了决定性的作用。

(二)狠抓交通基础设施建设，经济发展条件持续改善。我们坚持把交通作为基础设施建设的重点，围绕打造区域性交通枢纽城市，全力构建“五高三铁两江一机场”立体化交通框架，完成交通投资56.73亿元，增长74.1%。桂梧高速公路、八步至昭平二级公路八黄段建成通车，广贺高速公路贺州段即将全线贯通。洛湛铁路客货运已全面通车运营。永贺高速公路贺州段全面开工建设，贵广快速铁路贺州段全线施工。新客运中心交付使用。贺州至河池巴马高速公路、贺州至韶关高速公路贺州段、柳韶铁路贺州段和贺州机场等重大交通项目前期工作顺利推进。贺江、桂江已纳入西江“亿吨黄金水道”发展规划。

(三)扎实推进产业结构调整，工业主导地位不断增强。我们扎实开展“服务企业年”活动，帮助企业解决生产经营困难。财政安排企业扶持资金2115万元，安排融资担保配套资金500万元。我市率先在全区成立了第一家小额贷款公司，2009年全区共获批开业小额贷款公司23家，其中贺州占5家。及时召开银行企业项目对接会，着力解决中小企业融资难题，使停产半停产企业逐步恢复生产，工业生产在下滑通道中逐步企稳回升，全年新增规模以上工业企业16家。全力实施143项重点工业项目建设，完成投资80.15亿元，完成年度投资计划的104.4%。开工建设了桂东电子节能产品用储能材料技术改造、邝氏矿业公司陶瓷原料加工、大桂山50万立方米人造板生产线和新凯骅木业公司年产10万立方米高密度纤维板二、三期工程等重点工业项目，着力抓好华润贺州电厂、华润富川水泥厂、吉光电子等具有支撑带动作用的产业项目。加快旺高、西湾(平桂)、信都工业区和各县(区)工业集中区基础设施建设，启动贺州电子科技工业园规划建设。

(四)加快城镇化建设，城乡统筹发展有序推进。我们坚持把推进城镇化作为扩内需保增长的重点，完成了我市土地利用总体规划和城市总体规划的修编工作，并已上报自治区审批。启动实施国家森林城市创建工作，创建总体规划已通过评审。强化经营城市理念，多渠道筹措资金，加紧推进“一江两岸三新区”建设，快速推进南环路景观改造、堤路园、湖广大市场、经济适用房等项目建设，市垃圾填埋场一期工程投入使用，市污水处理厂投入试运营。公交线路、运营车辆不断增加，市政设施日趋完善。“大种树、种大树”活动成效初显，环境整治工程和城乡清洁工程深化拓展，城市管理水平得到提升。

(五)采取措施拓展市场，扩大消费取得明显成效。出台扩内需保增长八大举措，成功举办2009‘中国(贺州)瑶族盘王节暨广西贺州第五届农产品展销会，签约项目投资额和交易量大幅增加。落实家电汽车下乡、以旧换新政策，兑现家电下乡补贴资金1368.5万元，深入推进“万村千乡”市场工程和“新网工程”建设，新增“农家店”50家。房地产市场快速回暖，完成销售额6.13亿元，增长33%。以生态休闲为主体的旅游业加快发展。桂台(贺州)客家文化旅游合作示范区获自治区党委批复，大桂山国家森林公园旅游景区项目、黄姚古镇景区项目顺利实施，两广六市旅游合作深入推进，首趟旅游专列贺州游获得成功。新建20个乡村旅游示范点。全年接待游客423.4万人次，增长14.2%；旅游总收入27.6亿元，增长24.7%。

(六)切实加强“三农”工作，新农村建设迈出新步伐。扎实推进“五个一批”工程。全面落实各项惠农政策，惠农补贴兑现到户。村村通电工程新建配电台区136个，解决30147人用电困难。新建沼气池1.59万座，完成病险水库除险加固9座，农田水利基本建设、农村危房改造、安全饮水工程成效显著，农村生产生活条件明显改善。实施第三批41个贫困村整村推进扶贫开发。农村土地经营权流转规范有序，新增流转土地12.5万亩。耕种收机械化水平达27.2%。粮食总产量70.2万吨，增长4.3%。肉类总产量14.7万吨，增长7%。林改工作完成勘界669.51万亩，完成自治区下达年度任务的203.7%；林权证发证222.46万亩，完成自治区下达年度任务的100.2%。新增脐橙、茶叶、马蹄等优势农产品种植面积5万亩。农技推广扎实有效，富川瑶族自治县列入“全国农技推广体系改革与建设示范县”。农业产业化经营深入推进，新增规模农业企业32家、农民专业合作社25家。完成农民素质培训34.5万人次，农村劳动力实现转移就业4.5万人。

(七)深入推进体制改革，开放合作进一步扩大。完成国有企业改制3家，解决了一大批历史遗留问题。粮食购销企业产权制度、集体林权制度、医药卫生体制等改革取得积极进展。全面落实结构性减税和税费改革政策，全年减税5000万元，减轻企业负担。财政体制改革不断深化，财政支出结构调整优化，财政摆脱了徘徊局面，实现了恢复性增长。扩内需项目配套资金、“三农”、义务教育等重点支出需要得到有效保障。加快金融产业创新发展，引进桂林市商业银行在贺州设立支行。认真落实鼓励全民创业、加快中小企业发展各项政策措施，非公有制经济加快发展。面对严峻经济形势，积极创新招商思路，加强承接产业转移平台建设，加大招商引资工作力度，组团参加第六届中国-东盟博览会等招商引资活动，招商引资成效显著。引导外贸企业调整产品结构，开拓新市场，外贸出口率先在全区实现由负转正，增幅始终排全区前列。

(八)统筹经济社会协调发展，社会各项事业全面进步。扎实推进教育事业。市职教中心建设完成投资8532万元，占年度任务的189.6%；贺州第二高中、富川瑶族自治县高中顺利通过自治区示范性高中评估；投入7563万元实施中小学校舍安全工程，新建校舍6.77万平方米；贫困生资助管理体系进一步完善，累计发放补助金7014万元，资助贫困生78489人，实现了“不让一个学生因家庭贫困失学”的目标。公共卫生防疫体系不断健全，认真抓好手足口病、甲型H1N1流感防控和食品药品监督工作。投入9299万元建设县级医院和乡镇卫生院，县(区)、镇、村三级医疗卫生服务体系逐步完善。人口计生工作稳步发展，富川县被评为“全国计划生育优质服务先进单位”，我市在全自治区率先实现了“’国优‘和’省优‘先进县区总体达80%”的目标。对外宣传工作取得突破，文体、科技事业稳步发展。我市被评为“自治区文明城市”，昭平县被评为“全国文化先进县”和“全国文明县城”；全市建成农家书屋69家，版权保护工作获全国一等奖；完成第三次全国文物普查田野调查以及全市非物质文化遗产普查工作；新建65个村级篮球场，获广西年度各项锦标赛金牌49枚；第四轮科技创新计划扎实推进，科普惠农兴村计划深入实施，昭平县承担的科技部第一批科技富民强县项目通过验收，八步区、钟山县、昭平县通过全国县(市)科技进步考核。“五五”普法扎实推进，基层民主逐步加强。民族工作成绩斐然，市教育局和富川瑶族自治县被评为“全国民族团结进步模范集体”。加强国土资源管理，做好依法征地工作，全市重点项目累计完成征地46230亩，完成率达89.1%；拆迁房屋265816平方米，完成率达73.9%，有力地保证了项目建设用地需求。扎实开展“矛盾纠纷大排查、领导干部大接访、政策法律大宣讲”活动，信访来访批数和人次分别下降58.2%和53.1%，重大活动期间没有群众赴邕进京非正常上访。安全生产形势总体平稳。

(九)坚持为民办实事，民生得到保障和改善。十件民生实事顺利推进。火车站至西湾(平桂)工业基地一级路完成投资9000万元，站前大道路基基本形成；南环路景观改造工程即将完工，并继续向西环路推进；市政设施建设顺利实施；市污水处理厂投入试运营；完成乡乡通油路或水泥路工程，在全自治区率先实现全部乡镇通等级油路或水泥路的目标，乡乡通油路或水泥路通达率位居广西第1；村村通广播电视直播卫星覆盖工程完成自治区下达任务，农村广播、电视覆盖率分别达93.3%和98%；严打整治斗争深入开展，现行命案侦破率达94.5%，“两抢一盗”案件立案率增长193.3%、破案率增长77.48%；在全区率先完成乡镇级人口和计划生育服务机构标准化、规范化建设任务；残疾人关爱工程免费为贫困白内障患者实施复明手术724例，免费为贫困缺肢残疾人装配假肢180例；高校毕业生就业帮扶工程帮助2238人就业，就业率82.31%。帮扶城镇失业人员再就业4258人，社会保险累计扩面473559人，征收社保费4.12亿元。住房公积金制度覆盖率达80.75%，经济适用住房首期702户已摇号，廉租住房保障范围由上年284户784人增至1108户3306人。适当降低城镇职工和居民基本医疗保险住院起付标准，新型农村合作医疗参合率90.2%。城镇、农村低保实现动态管理下的应保尽保。农村药品“两网”建设和农村集体聚餐报告工作稳步推进。困难群众大病救助补偿和“五保”对象年供养标准分别提高到1万元和1440元。加强节能减排和生态环境保护，限期治理15家污染企业，加大饮用水源保护力度，环保“三同时”制度执行率达95%。

(十)加强政府自身建设，公共服务能力不断提高。深入开展学习实践科学发展观活动，政风行风不断改进。深化行政审批制度改革，取消一批审批事项，大力推进投资项目代办制，实行现场审批、联合审批，积极推行政府采购招投标制度。完善政府工作运行机制，修订政府工作规则，规范工作程序。完善行政决策机制，坚持重大事项向市委报告制度，市政府重大决策邀请市人大、政协领导参与讨论，定期向市人大报告、向市政协通报工作。全年办理人大代表议案60件、政协委员提案108件，办复率100%。深化政务公开，加大行政监察和审计工作力度，廉政建设进一步加强。加强与各民主党派、工商联和工会、共青团、妇联的联系。人事编制、政府法制、行政服务、统计、物价、质量监督、史志、档案、气象、老龄、残联、外事侨务、民族宗教、对台事务、人民防空、民兵预备役和国防动员等工作都取得了新的成绩。

各位代表，过去的一年，我们沉着应对来自各方面的严峻挑战，全力以赴保增长保民生保稳定保良好发展势头，既取得了显著的发展成果，又积累了在复杂困难的环境中推动经济社会又好又快发展的经验。一是始终把中央、自治区扩内需保增长一系列决策措施作为我市加快自我发展的重大机遇，全力争取上级在资金、项目、政策等方面给予更大力度的支持，办成了一批多年想办而未能办的大事难事。二是始终坚持党政一心，上下一致，不争论，不埋怨，同心协力，认真抓好各项工作，有效地保持了经济平稳较快增长。三是始终注重创新工作机制，认真落实“四个非常”，以超常规的措施和办法来推动和开展各项工作，组建了市重点项目建设指挥部，实施了“六个一”项目管理模式，创新了“三超常”项目推进机制、开展部门联合审批，有力地加快了重大项目建设，得到了自治区领导和有关部门的高度赞扬。四是始终保持奋发有为的精神状态，咬紧牙关、顽强拼搏、埋头苦干，化危为机，以务实的作风推进各项工作落实，使工作取得了实效。

各位代表，我们取得的成绩来之不易。这是全市人民齐心协力、艰苦奋斗的结果，也是社会各界关心帮助和大力支持的结果。在此，我代表市人民政府，向辛勤工作在各条战线的广大干部群众，向给予政府工作大力支持的人大代表和政协委员，向驻贺部队、武警官兵和政法干警，向各民主党派、工商联、人民团体和社会各界人士，致以崇高的敬意和衷心的感谢！

在肯定成绩的同时，我们也清醒地看到，我市经济社会发展中仍存在不少困难和问题：经济结构性矛盾突出，经济总量及部分经济指标增速在全区排名靠后；部分重点项目进展不理想，部分企业经营困难，财政增收难度加大；就业、维稳工作压力增大，社会治安、安全生产形势严峻；一些涉及群众切身利益的问题还没有解决好，改善民生任务繁重；政府职能转变还不够到位，服务效能有待提高。对此，我们一定高度重视，采取有效措施，在新的一年里努力加以解决。

二、2010年工作总体要求和主要目标

2010年，是实施科学发展突破发展三年计划和“十一五”规划的最后一年，也是在复杂形势中巩固扩大发展成果的关键一年。我们既面临严峻挑战，更有重大机遇。当前，国际金融危机的影响依然存在，经济环境中不确定因素较多，支撑经济增长的内生动力仍然不足。同时，我们也要看到，国家应对危机一揽子政策效应继续显现，经济回升向好的基础逐步巩固，国家继续实施积极的财政政策和适度宽松的货币政策，也更加有利于我们争取国家投资实现更快的发展。近年来我们依托良好的区位、资源和产业等综合优势，扎实推进“四大突破”，各项重点工作取得显著成绩，也为保持发展良好势头提供了有力支撑。因此，我们既要增强忧患意识，又要坚定发展信心，积极主动地做好应对各种困难和挑战的准备，努力实现全市经济社会又好又快发展。

2010年政府工作的总体要求是：全面贯彻党的十七大和十七届三中、四中全会以及中央、全区经济工作会议精神，深入贯彻落实科学发展观，紧紧抓住中央继续实施积极的财政政策和适度宽松的货币政策以及国家实施新一轮西部大开发和中国-东盟自由贸易区建成的机遇，加快推进交通、工业、城建、旅游四大突破发展，特别要突出加快城市建设，着力提高经济增长质量和效益，着力转变经济发展方式和调整经济结构，着力深化改革开放和机制创新，着力改善民生和保持社会和谐稳定，努力实现科学发展突破发展三年计划和“十一五”规划目标，促进经济社会又好又快发展。

2010年全市经济社会发展的主要预期目标是：生产总值增长12%，财政收入增长12%，全社会固定资产投资增长30%，社会消费品零售总额增长16%，外贸进出口总额增长20%，万元生产总值能耗降低3%，二氧化硫排放量控制在3.98万吨以内，化学需氧量排放量控制在3.2万吨以内，城镇居民人均可支配收入增长12%，农民人均纯收入增长12%，城镇登记失业率控制在4.5%以内，人口自然增长率控制在9‰以内。

三、2010年主要任务

实现今年经济社会发展目标，必须着重抓好九个方面工作：

(一)着力扩大投资规模，增强投资对经济的拉动力

全力推进项目建设。继续保持较高的投资增幅，发挥投资对经济增长的强劲拉动。要加快推进重大项目新开工。今年安排1000万元以上的新开工项目97个，总投资205亿元，年度计划投资54亿元。继续加快续建项目施工进度，努力完成更多的投资量。今年安排1000万元以上的续建项目175个，总投资519亿元，年度计划投资129亿元。要把能源和交通基础设施建设作为完成投资目标任务的主战场，加快华润贺州电厂、永贺高速公路和贵广快速铁路贺州段、黄姚至昭平二级路等项目建设。继续抓好前4批中央投资项目的续建和新下达计划投资项目的新建工作，确保资金配套，确保施工质量，确保按期完成。

加强项目协调服务。继续实施“项目建设年”活动，继续由市重点项目建设指挥部统筹推进项目建设，落实重点项目领导联系制度，推行“六个一”项目管理模式等行之有效的项目推进机制，加快项目建设进度。进一步完善部门项目集中联合审批制度，建立并联审批工作机制，提高审批效率。充分发挥各职能部门作用，开展帮扶活动，帮助解决项目用地、审批、环评、资金等突出问题，促进更多项目开工建设和竣工投产。

做好项目前期工作。今年市本级安排1500万元项目前期经费，各县(区、管理区)也要加强前期工作经费保障。要组织力量编制好“十二五”规划，并结合规划编制，按照中央投资方向和重点，谋划和储备一批项目，加强与上级有关部门的汇报衔接，加快项目申报审批进度。要抓紧编报中央扩内需投资项目计划，争取更多的中央投资。加快推进贺州至河池巴马高速公路、贺州至韶关高速公路、柳韶铁路贺州段、贺州机场等重大项目前期工作。

(二)着力加快产业优化升级，提高工业经济质量和效益

大力发展优势产业。进一步调整结构、改造提升、提高效益，争取在“十二五”期间把电力、林产、矿业、电子四大优势产业打造成为百亿元产值的产业，发展上下游产业，形成产业链。加大技术改造投入力度，组织实施280个总投资120亿元的新建、续建技改项目，确保技改投资增长20%以上。培育发展后续支撑产业，推动华润富川水泥厂、林浆纸一体化、林板一体化等重大项目早日建成投产。力争工业增加值突破135亿元，增长12%。

培育壮大骨干企业。深入实施“中小企业成长工程”，力争新增10家规模以上企业。支持桂东电子、灵峰药业、金源稀土、平桂飞碟等成长型企业发展，通过资产重组、资源整合、上市融资，培育一批年销售收入超10亿元的龙头企业，带动一批配套中小企业集群发展，提升优势特色产业整体竞争力。

突出发展产业园区。抓好旺高、西湾(平桂)、信都工业区和各县工业集中区建设，加快筹建华润(富川)生态循环产业园区，争取贺州电子科技工业园列入自治区“十二五”规划并启动建设，力争各类工业园区完成基础设施投资5.21亿元，增长8%。完善园区管理体制和运行机制，加大政策扶持力度，积极探索市场化开发路子，加快园区内水电路等基础设施建设，提升产业转移承载能力，促进新增工业项目向园区集聚。创新园区招商思路，着力创建承接产业转移示范区。

提高自主创新能力。完善科技创新体系，全面提升企业技术创新能力。认真实施第四轮创新计划，围绕电子、林产、有色冶炼、石材、农产品加工等支柱产业，逐步实施并完成一批科技攻关项目，开发高端配套产品。继续以“人才小高地”为载体，加快培养、吸引和集聚高层次创新新型人才。

加强节能减排工作。强化节能减排目标责任制，抓好重点行业和重点企业的节能减排，大力发展低碳经济。加大高耗能高污染落后产能淘汰力度，对限期淘汰的落后装备严格监管。

(三)着力推进城镇化建设，促进城乡一体化发展

全力推进城市建设。全面实施城市基础设施建设三年大会战，组建市城建重点项目指挥部，推进总投资50亿元的城市建设重点工程项目建设，加快“一江两岸三新区”建设，深化“贺八平钟”一体化进程，扩大城市规模，完善城市功能，增强综合承载能力。完成火车站至西湾(平桂)工业基地一级路、火车站货场路、望高火车站进站路、贺州至旺高工业区一级路一期工程、贺州至姑婆山一级路、环城一级路、城东新区主路网等城市路网工程项目的建设。整合国有集体资源，组建贺州市城市投资开发集团公司，做强做大融资平台，做好经营城市文章。

完善城乡建设规划。加快城市总体规划实施，完成贺州市绿地系统和给排水系统专项规划，制定城乡建筑风貌改造规划、城市高楼规划以及城市规划区农民建房规划和城中村改造规划。支持平桂管理区完善总体规划，加快平桂管理区行政中心建设。及时修编一批乡镇总体规划和乡镇土地利用总体规划，分批编制村庄规划，建立覆盖城乡的规划管理体系。

加强城乡环境整治。建立健全城乡国土、建规执法体系，改革执法机制，加大对城乡违法用地、违章建房的查处力度。巩固“城乡清洁工程”成果，实施城镇净化、绿化、亮化、美化工程。县级污水处理厂今年6月底前建成运营，各县垃圾处理厂年底前建成运营，全市城镇污水集中处理率和垃圾无害化处理率达60%以上。加大市区排洪河整治力度。全面实施城乡风貌改造工程，以改造“竹筒房”为重点，结合城乡市政公共设施配套建设和农村住房建设改造，改善城乡人居环境。

发展壮大县域经济。加快推进城镇化，进一步放宽城镇户籍限制，鼓励符合条件的农业人口转移到城镇就业和落户，大力发展农村二、三产业，多渠道推动农民持续增收。扶持贫困山区加快发展，在项目、资金安排等方面给予倾斜，在交通、水利等基础设施建设上给予适当补助。立足比较优势，大力发展特色优势产业，培育壮大一批经济强县、经济强镇。

(四)着力夯实农业基础地位，促进农村经济稳定发展

加强农村基础设施建设。继续实施新农村建设“五个一批”工程，着力改善农村生产生活条件。完善财政支农投入稳定增长机制，确保财政支农投入增量高于上年。整合资源，实施“村村通”等级路工程，新修改造农村公路270公里；新建农村沼气池1.73万座；加强病险水库除险加固和灌区水利工程及配套设施建设。加大对库区移民和自然保护区群众的生产扶持。

推进农业产业化经营。抓好农业产业调整，稳定粮食生产，确保粮食总产量达71万吨，大力发展水果、蔬菜、茶叶、烟叶等高效生态特色农业。继续抓好林业生态建设，完成495.36万亩林改任务。做大做强畜牧水产业，推动畜牧水产业规模化、标准化养殖，支持温氏畜禽养殖公司、巨东养殖公司等企业大力推行“公司+基地+农户”的养殖发展方式。加强农作物病虫害的防控，强化动物疫病防控和畜产品安全体系建设。扶持发展农业龙头企业，新增龙头企业5家，产业化经营带动农户面达35%。推进土地适度规模经营和土地承包经营权依法有序流转，完善农业技术服务体系，推进农业政策性保险。

多渠道增加农民收入。认真落实各项惠农强农政策，足额兑现各项补贴政策。继续推进第三批41个贫困村整村推进扶贫开发。注重抓好大宗农产品销售工作，逐步建立农产品直销市场，搞活农产品流通，加快发展农产品加工业，促进农民就业创业增收。支持发展多种形式的农民专业合作经济组织，完善利益联结机制，提高农业生产组织化程度。大力发展劳务经济，培训农民工3.36万人次，劳务输转3.3万人次。

(五)着力扩大消费需求，增强消费拉动力

做大做强旅游业。启动建设桂台(贺州)客家文化旅游合作示范区。加强旅游投融资平台建设，引资加快黄姚古镇景区、大桂山国家森林公园旅游景区项目、姑婆山国家森林公园二期工程、“桂江百里竹海”、二十里贺江竹廊、月湾茶园等景区景点以及乡村旅游示范点建设。加强旅游宣传促销，打造“桂东北山水精华游”精品旅游线路。深化区域旅游合作，扩大两广六市旅游合作领域。加强旅游行业管理，完善旅游接待设施，提升接待服务水平。力争接待游客人数增长10%，旅游总收入增长12%。

大力发展服务业。大力发展商贸流通、餐饮、住宿等传统消费性服务业，引导商贸服务企业合理布局，加强汽车交易、蔬菜批发、建材市场、小商品批发等专业市场建设。发挥供销合作社的主导作用，促进农村商品流通。深入推进“万村千乡”市场工程和农村新型流通网络建设，激活农村消费市场。力争村级农家店覆盖率达100%。加快发展现代物流业，完善物流配送体系，加快贺州火车站物流中心建设，提升华安汽运等物流企业的服务功能。积极发展咨询、评估、法律、会计等中介服务和保险、证券等金融服务。

扩大居民消费需求。认真落实好国家鼓励消费的财税、金融政策，深入推进家电、汽车、摩托车下乡以及家电、汽车以旧换新工作。积极发展房地产业，完善住房消费的税收、信贷政策，支持居民自住和改善性购房需求。鼓励县城和重点镇发展房地产业，引导农村劳动力进城安居就业。加大农村危房改造力度。努力增加城乡居民特别是中低收入者的收入，增强居民即期消费能力。稳步扩大餐饮、商贸传统消费，积极培育旅游、文化、健身等新兴消费热点。扩大和完善消费信贷，加强市场监管监测，保持物价基本稳定，营造良好的居民消费环境。

(六)着力深化改革开放，增强经济增长活力

深入推进各项改革。深化国有企业改革，突出抓好重点企业改制后的二次招商和资产重组，妥善解决企业改制后的历史遗留问题。加快粮食、商贸等流通领域企业的改革步伐。完善农村公益性服务机制，深化集体林权制度以及医疗卫生、财税金融、文化教育体制和事业单位等方面的改革。加快行政管理体制改革，全面完成政府机构改革任务。认真落实鼓励全民创业各项政策，鼓励和支持非公有制经济进入基础设施、公用事业、金融服务和社会事业等领域，着重推进民营企业与其他所有制企业在投资审批、土地、外贸、财税扶持等方面的待遇公平化，营造公平竞争的市场环境。

加大招商引资力度。组团参加第六届泛珠经贸洽谈会、桂台经贸合作交流会、广州中小企业博览会、第七届中国-东盟博览会等招商引资活动，组织小分队到东南亚、港澳台、长三角、珠三角地区“走出去”招商，实行重点投资商“请进来”考察，引进更多的优势资源和产业链招商项目。不断拓展区域合作领域，完善投资优惠政策，严格落实招商引资考核办法，深入开展招商引资项目大兑现工作，提高招商引资的规模和质量。力争全年引进内资项目到位资金增长15%，实际利用外资到位资金增长20%。

努力扩大外贸出口。积极应对外贸出口新形势，优化出口商品结构，巩固钛白粉、机电产品、食品、冷冻蔬菜等特色产品的出口优势。加大对外贸企业发展政策扶持力度，充分用好用足国家政策，进一步拓展国际市场，推动外贸出口稳定增长。

(七)着力抓好财政金融工作，提高发展保障能力

促进财政稳定增长。积极培植财源壮大财力，力争财政收入突破20亿元。积极筹措和安排融资担保配套资金，重点推进城市基础建设和重点项目建设，增强项目建设实现税收对财政收入的拉动作用。积极争取落实技术改造和科技创新资金，支持企业技术改造和科技创新。整合支农资金，实施为农民办实事工程和以农田水利设施为主的农业基础设施建设。转变财政投资方式，调整财政投资策略，把有限的财政资金作为壮大财政综合实力的资本来运作，提高财政资金使用效益。

坚持依法理财治税。严格综合预算，以“金财”工程为依托，加大非税收入管理，把政府公共资源有偿使用招标、拍卖等收入纳入预算管理，将行政事业单位的国有资产出租收入和处置收入纳入“收支两条线”管理。抓好重点税源以及车船税、城镇土地使用税、房产税、资源税等零散税收的征管。全面推广税控收款机的应用，规范旅馆、餐饮、服务娱乐等行业的税收管理。从严控制一般性支出，在公务购车和用车经费、会议经费、公务接待费用、党政机关出国(境)经费预算方面继续实现零增长， 暂停一切楼堂馆所建设的预算开支。

强化金融保障服务。积极推动驻贺金融机构升格和“引银入贺”工作，抓好农村金融改革试点工作。成立市金融办，争取获批开业3家小额贷款公司。实行信贷投放激励机制，推进政企、银企合作，引导商业银行增加信贷投放，缓解中小企业融资难问题。金融机构要把握政策机遇，进一步创新和开发金融产品。要加强金融监管，防范金融风险。

(八)着力保障和改善民生，推进和谐社会建设

千方百计扩大就业。稳定现有就业岗位，多渠道拓宽就业门路。全面落实就业有关政策，鼓励高校毕业生到基层工作和自主创业，加强对农民工流动就业的引导和服务，积极支持农民工返乡创业。积极做好复转军人安置工作。完善城乡就业服务和援助机制，加大帮扶力度，加强职业技能培训，以创业带动就业，重点做好就业困难人群、零就业家庭就业援助工作。

完善社会保障体系。积极扩大城镇社会保险覆盖面，使养老、医疗、失业、工伤和生育等社会保险覆盖各类企业。探索城乡老年居民、农民工等群体的养老保障办法。(下转第四版)（上接第三版）健全城镇职工医疗保障体系，城镇居民医疗保险覆盖面达90%以上，巩固完善新型农村合作医疗制度，解决好群众看病难、看病贵问题。进一步提高企业退休人员养老金水平和城乡居民最低生活保障标准。落实低收入群体帮扶救助措施，保障困难群众基本生活需要。维护妇女、未成年人、老年人和弱势群体合法权益，重视发展慈善、社会福利、老龄和残疾人事业，做好红十字会工作。

发展各项社会事业。坚持优先发展教育。加强教育资源统筹，促进教育均衡化发展；扎实推进校舍安全工程和教育质量提升工程；做好留守儿童教育管理工作；加快市职教中心建设，全面完成职教攻坚任务；加快推进贺州学院二期工程建设，积极筹建贺州学院附属高中并实现开班办学。积极发展文化、新闻出版、体育、广播电视事业。加强城乡公共文化设施建设，新建19个乡镇综合文化站、131家农家书屋。强化城乡社区卫生服务，提高医疗卫生服务水平。全面落实农村计划生育家庭奖扶政策，推行村级人口和计划生育工作常态化管理，巩固和扩大创优成果，争取在全区率先实现全国计划生育优质服务先进县“满堂红”。抓好第六次全国人口普查。继续加强国防教育和国防动员建设，扎实做好人民武装、民兵预备役和“双拥”工作。深入实施《全民科学素质行动计划纲要》，不断提升全民科学素质。加强基层民主法制建设，推进普法教育。继续加强公民思想道德教育和精神文明建设。支持工会、共青团、妇联开展工作。认真落实党的民族、宗教和侨务政策，进一步抓好外事、对台、史志、档案、审计、物价、气象、人民防空、防震减灾等工作。

加强生态环境建设。推进国家森林城市和全区生态文明示范城市创建工作，大力开展造林绿化，突出抓好高速公路、铁路等通道和主要河流河段以及城区、重点城镇、乡村绿化。继续巩固退耕还林成果，实施石漠化治理。加强重点流域、水源保护区的综合治理。强力实施“贺江清新行动”，加强保护龟石水库水资源，缓解水源污染问题，不断改善城乡环境质量。

全力维护社会稳定。继续开展“矛盾纠纷大排查、领导干部大接访、政策法律大宣讲”活动，有效预防和化解矛盾纠纷。深入推进“社会和谐稳定模范市”创建活动，完善社会治安防控体系，依法严厉打击违法犯罪活动。落实安全生产责任制，强化重点领域、重点行业的专项整治及有效监控，坚决遏制重特大安全生产事故。强化产品质量和食品药品安全监管。完善应急预案体系，提高应对突发公共事件的能力。

坚持为民办实事。从解决群众最关心、最直接、最紧迫的问题入手，坚持每年为群众办一批实事。继续实施20户以上村村通电工程，解决149个自然村32464人用电问题。全面完成20户以上“村村通”村点直播卫星覆盖工程和乡镇农村广播电视网络光纤联网工程。继续推进农村危房改造、安全饮水工程和廉租住房、经济适用房建设等工作。

(九)着力加强政府自身建设，提高行政能力和服务水平

全面推进依法行政。主动接受人大的法律监督和政协的民主监督，主动听取民主党派、工商联、人民团体和社会各界人士的意见和建议，提高议案、建议和提案办理质量。不断完善行政决策程序，建立行政决策纠错机制，加大违法决策责任追究力度。严格执行规范性文件制定和备案制度。深化行政执法体制改革，规范行政执法行为，努力打造法治型政府。

努力提高行政效率。完善经济目标和机关效能建设目标绩效考评机制，切实改进工作作风，坚持深入基层帮助解决实际问题，大力整顿会风文风，加强督促检查，狠抓工作落实。完善联合审批制度，推进行政审批项目标准化工作。深入推行首问负责制、限时办结制和责任追究制，做到服务事项规范统一，服务程序公开简化，办事时限压缩提速，努力打造服务型政府。

着力加强廉政建设。加强政府采购招投标、重大工程建设、财政性资金和社会公共资金的监督管理，强化行政监察和审计监督。着力纠正损害群众利益的不正之风，严肃查处涉企“三乱”行为。严格落实廉洁自律、厉行节约的各项规定，着力完善惩防体系，努力打造廉洁政府。

提高改革创新能力。要加强事关经济社会发展的新理念、新举措以及工业化、城镇化等方面的政策和知识的学习，认真研究新情况，积累新经验，不断提高抢抓机遇的能力、创新发展的能力、依法行政的能力、高效落实的能力和破解难题的能力，努力打造学习型政府。

各位代表！贺州发展正处在一个关键时期，困难和挑战考验着我们，责任和使命激励着我们。让我们在市委的坚强领导下，坚定信念，攻坚克难，开拓创新，扎实工作，为实现贺州科学发展突破发展而努力奋斗！
